# Association between fibroblast growth factor 19 and depressive symptoms: the moderating role of smoking

**DOI:** 10.1017/neu.2025.10028

**Published:** 2025-07-14

**Authors:** Siyuan Li, Lingling Chen, Mingwei Ma, Yueling Hu, Fan Wang, Xingguang Luo, Yu-Hsin Chen, Hongke Gao, Yulin Ren, Weiming Hu, Yimin Kang, Wei Wang, Li Chen, Xiaokun Li, Yanlong Liu, Junnan Wu

**Affiliations:** 1 School of Mental Health, Wenzhou Medical University, Wenzhou, China; 2 Beijing Hui-Long-Guan Hospital, Peking University, Beijing, China; 3 Department of Psychiatry, Yale University School of Medicine, New Haven, CT, USA; 4 The Third Hospital of Quzhou, Quzhou, Zhejiang, China; 5 Psychosomatic Medicine Research Division, Inner Mongolia Medical University, Hohhot, China; 6 School of Pharmaceutical Science, Wenzhou Medical University, Wenzhou, China; 7 Department of Pharmacy, The Quzhou Affiliated Hospital of Wenzhou Medical University, Quzhou People’s Hospital, Quzhou, China

**Keywords:** Fibroblast growth factor 19, smoking, Beck’s Depression Inventory scores, cerebrospinal fluid, depressive symptoms

## Abstract

**Objective:**

This study aimed to examine the relationship between fibroblast growth factor 19 (FGF19) and depressive symptoms, measured by Beck’s Depression Inventory (BDI) scores and investigate the moderating role of smoking.

**Methods:**

This study involved 156 Chinese adult males (78 smokers and 78 non-smokers) from September 2014 to January 2016. The severity of depressive symptoms was evaluated using the BDI scores. Spearman rank correlation analyses were used to investigate the relationship between cerebrospinal fluid (CSF) FGF19 levels and BDI scores. Additionally, moderation and simple slope analyses were applied to assess the moderating effect of smoking on the relationship between the two.

**Results:**

FGF19 levels were significantly associated with BDI scores across all participants (*r* = 0.26, *p* < 0.001). Smokers had higher CSF FGF19 levels and BDI scores compared to non-smokers (445.9 ± 272.7 pg/ml vs 229.6 ± 162.7 pg/ml, *p* < 0.001; 2.7 ± 3.0 vs 1.3 ± 2.4, *p* < 0.001). CSF FGF19 levels were positively associated with BDI scores in non-smokers (*r* = 0.27, *p* = 0.015), but no similar association was found among smokers (*r* = −0.11, *p* = 0.32). Linear regression revealed a positive correlation between FGF19 and BDI scores (*β* = 0.173, *t* = 2.161, 95% CI: 0.015–0.331, *p* < 0.05), which was negatively impacted by smoking (*β* = −0.873, *t* = −4.644, 95% CI: −1.244 to −0.501, *p* < 0.001).

**Conclusion:**

These results highlight the potential role of FGF19 in individuals at risk for presence of or further development of depressive symptoms and underscore the importance of considering smoking status when examining this association.

Significant outcomes

1. Smoking modulates the relationship between FGF19 and depressive symptoms as a moderator.
2. Smokers have higher CSF FGF19 levels and BDI scores compared to non-smokers.
3. Participants with higher BDI scores have higher CSF FGF19 levels.

Limitations

1. This study has limitations in generalizability, as the sample consisted exclusively of Chinese adult males. Future studies should include more diverse populations (e.g., females, other ethnic groups) to enhance external validity.
2. Although adjustments were made for several demographic and lifestyle factors (e.g., age, BMI, marital status), residual confounding due to unmeasured variables (e.g., genetic predispositions, comorbid metabolic or psychiatric conditions) cannot be ruled out.
3. Additionally, potential recall bias related to self-reported smoking behaviour and depressive symptoms (BDI scores) may have affected the reliability of the data. Future studies should consider incorporating objective biomarkers (e.g., serum cotinine levels for smoking, clinician-rated depression scales) to strengthen measurement accuracy.

## Introduction

Mental disorders are a major contributor to the global health-related burden, and depressive symptoms are a major contributor to this burden (Monroe & Harkness, 2022), which has a significant impact on quality of life (Tran *et al*., 2020). The main manifestations of depressive symptoms include changes in somatic symptoms, negative affect and anhedonia, which can lead to significant personal and social burdens (Wojnowski & Zimmer, 1997). In addition, with an increase in many risk factors, such as economic stress and social isolation, it can temporarily lead to an increase in depressive symptoms (Laarne *et al*., 2000). Given the high prevalence of depressive symptoms and its significant impact on quality of life, research regarding early detection and intervention of emerging depression symptoms is warranted.

The neurotrophic hypothesis suggests that the neurobiological basis of mood disorders may be due to dysregulation of neurotrophic factors and their effects on brain circuits, which can cause a range of depressive symptoms (Xu *et al*., 2021). The underlying mechanisms of the antidepressant effects of drugs may also be related to the modulation of multiple neurotrophic factors (Castrén & Monteggia, 2021; Wang *et al*., 2022). Fibroblast growth factor (FGF) belongs to a large family of growth factors involved in brain development at an early age and in maintenance and repair throughout adulthood (Xu *et al*., 2021). Recent studies have suggested new roles for FGF members in depression (Turner *et al*., 2006; Lang & Borgwardt, 2013; Deng *et al*., 2019).

Fibroblast growth factor 19 (FGF19) is a circulating hormone that regulates a wide range of biological functions, including energy homeostasis and brain development (Beenken & Mohammadi, 2009; Gadaleta & Moschetta, 2019). In a cross-sectional study, altered levels of FGF19 and FGF21 were found to be common causative mechanisms for metabolic and cognitive deficits in patients with major depressive disorder (Tang *et al*., 2023). Moreover, FGF19 has been shown to be involved in cell proliferation and survival during embryonic brain development (Somm & Jornayvaz, 2018). Our previous study demonstrated a significant correlation between human cerebrospinal fluid (CSF) FGF19 levels and Beck Depression Inventory scores (Liu *et al*., 2017).

Smoking is more common among people with mental health problems (Lawrence *et al*., 2009; McClave *et al*., 2010; Wootton *et al*., 2020), especially those with depressive symptoms (Hall *et al*., 1993). Multiple studies have found that smoking increases the risk of depressive symptoms (Coultas *et al*., 2007; Hooshmand *et al*., 2012). As we all known, people with depression are more likely to be smokers (Richards *et al*., 2013; Fluharty *et al*., 2017; Li *et al*., 2022) and nicotine dependent (Dierker & Donny, 2008; Sweitzer *et al*., 2008). However, depressive symptoms are often exacerbated in people who quit smoking (Gravely-Witte *et al*., 2009). Tobacco contains substances such as nicotine, which primarily stimulates the brain. Nicotine acts mainly on nicotinic acetylcholine receptors (nAChRs), stimulating the release of norepinephrine, serotonin, dopamine, acetylcholine, gamma-aminobutyric acid, and glutamate in the brain (Haustein *et al*., 2002; Picciotto *et al*., 2002).

Until now, the association between FGF19 and smoking has been reported in some studies. For example, FGF19 has been implicated as a potential driver gene in laryngeal squamous cell cancer (LSCC) with clinical characteristics linked to smoking (Tan *et al*., 2016). However, the direct relationship between smoking and FGF19 is not yet known. Moreover, whether smoking plays an important role in the relationship between FGF19 and depressive symptom remains unclear, although most studies suggest that smoking itself increases the risk of depression (Makarov, 1973; Orth, 2002). Therefore, based on previous studies, the aim of the present study is to explore the association between depressive symptom and FGF19 in the CSF of smokers and non-smokers by assessing Beck’s Depression Inventory (BDI) scores.

## Materials and methods

### Participants

This cross-sectional analysis included 191 Chinese adults. After excluding participants with missing data, family history of psychiatric or neurological disorders, or systemic/CNS diseases (diagnosed via the Mini International Neuropsychiatric Interview), a total of 156 participants were included. Demographic and lifestyle data (age, BMI, marital and living status) were collected. Additionally, CSF samples and relevant clinical data were collected. Smoking status, including age of smoking initiation, daily cigarette consumption, smoking duration, and Fagerström Test for Nicotine Dependence (FTND) scores, was also recorded (Ríos-Bedoya *et al*., 2008). Smokers were defined per WHO criteria (≥ 1 year smoking history), and individuals with other substance use disorders were excluded. Non-smokers had no history of tobacco or other substance use. All participants provided written consent, and the procedures followed the ethical standards of the institutional anglasd/or national research committee, consistent with the 1964 Helsinki Declaration.

### Biosample collection and laboratory tests

CSF samples were collected following established protocols, as described previously in detail (Li *et al*., 2018). The levels of FGF19 in CSF were quantified using ELISA kits (Nanjing Jiancheng Bioengineering Institute, Nanjing, China) as per the manufacturer’s instructions (Xu *et al*., 2019). Double-blind principles were applied throughout the process.

### Assessment of depressive symptoms

The Beck’s Depression Inventory-II (BDI-II) was used, consisting of 13 items scored from 0–3. A total score > 4 indicated depressive symptoms, with scores categorised into mild (5–7), moderate (8–15), and severe (≥ 16). Assessments were conducted one day prior to CSF Collection.

### Statistical analysis

Continuous variables were compared using independent t-tests or Wilcoxon rank-sum tests; categorical variables were assessed with χ^2^ tests. Spearman correlations were used to examine associations between FGF19 and BDI scores across the full sample and subgroups (smokers vs. non-smokers). Multiple linear regression and moderation analyses (including interaction terms) were conducted with adjustments for age, BMI, marital and living status. All analyses were performed in R (v4.3.0), with statistical significance set at *p* < 0.05 (two-tailed).

## Results

### Population characteristics

In our sample, 63 individuals had BDI scores less than 1, while 93 individuals scored 1 or higher. Notably, 19 participants scored ≥ 5; among them, 5 had scores below 8, and 14 had scores between 8 and 16 (please refer to Supplementary Table 1).The study included 156 participants, equally divided into smokers and non-smokers. Significant differences in age, BMI, marital status, and living status were observed (*p* < 0.05). Smokers exhibited higher CSF FGF19 and BDI scores than non-smokers (445.9 ± 272.7 pg/ml vs. 229.6 ± 162.7 pg/ml, *p* < 0.001; 2.7 ± 3.0 vs. 1.3 ± 2.4, *p* < 0.001). No significant differences were observed in blood pressure (*p* > 0.05). For detailed demographics and biochemical indicators, please see Table 1. Additionally, we have included a new Supplementary Table 2, which presents the median and IQR for non-normally distributed variables to improve transparency and data interpretation.

**Table 1.** Comparisons between non-smokers and smokers

Characteristic	Non-smokers	Smokers	*p*-value^2^
	*N* = 78^1^	*N* = 78^1^	
**Demographics**			
Age*	29.8 (9.9)	33.7 (10.0)	0.014
BMI*	25.0 (4.2)	25.8 (3.6)	0.036
SBP	130.2 (12.7)	126.9 (13.1)	0.108
DBP	75.0 (8.9)	76.2 (11.4)	0.455
**Marriage**			0.009
Married	39 (50%)	23 (29%)	
Unmarried	39 (50%)	55 (71%)	
**Living status**			< 0.001
Living in family	56 (72%)	73 (94%)	
Living with others	22 (28%)	5 (6.4%)	
**Smoking**			
FTND score*		3.3 (2.2)	
Smoking onset*		20.0 (3.8)	
Smoking period*		13.4 (8.9)	
**Biochemical indicators**			
FGF19 (pg/ml)*	229.6 (162.7)	445.9 (272.7)	< 0.001
**Depression**			
BDI scores*	1.3 (2.4)	2.7 (3.0)	< 0.001

*Data with non-normal distribution.

*Note*: *p*-values for comparisons between smokers and non-smokers were calculated using the Chi-square test for categorical variables and the Wilcoxon rank-sum test for continuous variables.

**Abbreivation:** FGF19, fibroblast growth factor 19; BMI, body mass index; SBP, systolic blood pressure; DBP, diastolic blood pressure; BDI, Beck’s Depression Inventory-II; FTND, Fagerström Test for Nicotine Dependence; Smoking onset, age at smoking initiation (years).

### FGF19 and BDI correlations

**Figure 1.**
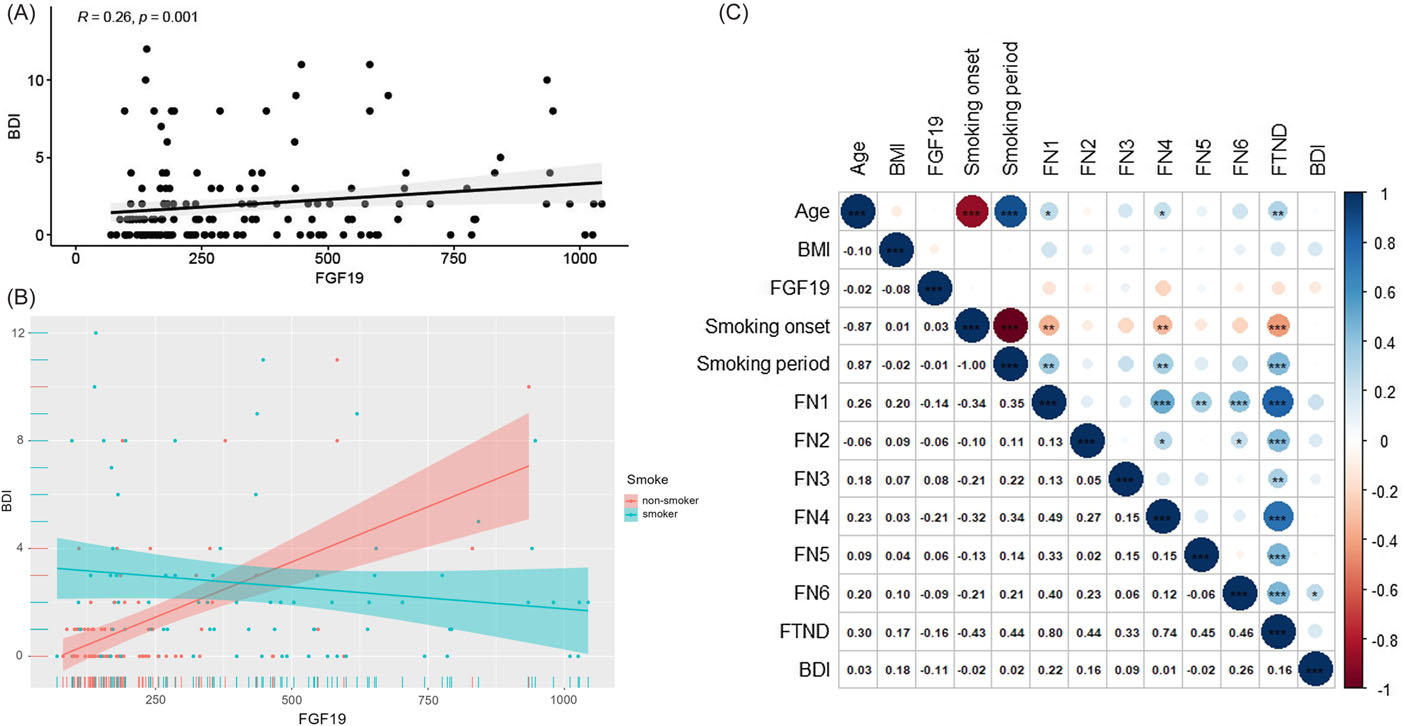
Correlation analysis between FGF19 and BDI scores. *Note*: (A) Spearman correlation analysis between FGF19 levels and BDI scores in all participants. (B) Bivariate correlation matrix using Spearman’s rank correlation for study variables in non-smokers and smokers. (C) Spearman correlations between internal smoking-related indicators and depressive symptoms in smokers.*p < 0.05, **p < 0.01, ***p < 0.001.In Panel C: left-side numbers indicate Spearman correlation coefficients. Blue circles represent positive correlations; red circles indicate negative correlations. Darker shades indicate stronger absolute correlation values.**Abbreivations:** FGF19, fibroblast growth factor 19; BMI, body mass index; BDI, Beck’s Depression Inventory; FTND, Fagerström Test for Nicotine Dependence; FN, FTND item scores; Smoking onset, age of smoking initiation (adjusted for age).

The level of FGF19 was significantly associated with BDI scores across all participants (*r* = 0.26, *p* < 0.001) (Figure 1A). FGF19 levels in CSF were positively associated with BDI scores in non-smokers, but no similar result was found among smokers (*r* = 0.27, *p* = 0.015; *r* = −0.11, *p* = 0.32) (Figure 1B). FTND scores positively correlated with years of smoking (*r* = 0.44, *p* < 0.001), and negatively with age at smoking initiation (*r* = −0.43, *p* < 0.001), adjusted for age (see Figure 1C).

### Moderation analysis

Based on the correlations in Figure 1, we further explored the inhibitory effect of smoking on the relationship between FGF19 and depressive symptoms severity using moderation analysis, adjusted for age, living status, BMI, and marital status (Table 2).

**Table 2.** Linear regression table for the moderation analysis

	Model 1			Model 2			Model 3		
	***β***	**95%CI**	***t***	***β***	**95%CI**	***t***	***β***	**95%CI**	***t***
Age	0.015	[−0.206, 0.236]	0.135	0.012	[−0.206, 0.232]	0.11	0.054	[−0.152, 0.260]	0.52
BMI	0.119	[−0.043, 0.280]	1.454	0.092	[−0.067, 0.252]	1.141	0.096	[−0.054, 0.247]	1.265
Marriage	0.271	[−0.179, 0.722]	1.19	0.222	[−0.225, 0.669]	0.981	0.072	[−0.353, 0.497]	0.335
Living	0.026	[−0.479, 0.531]	0.102	0.075	[−0.430, 0.580]	0.294	0.083	[−0.391, 0.556]	0.345
FGF19	0.173*	[0.015, 0.331]	2.161	--	--	--	0.741***	[0.424,1.058]	4.618
Smoke	--	--	--	0.444**	[0.120, 0.769]	2.708	0.195	[−0.146, 0.537]	1.129
FGF19* Smoke	--	--	--	--	--	--	−0.873***	[−1.244, −0.501]	−4.644
Adj. R^2^	0.033			0.049			0.167		
F	2.062 (5,150)			2.615 (5,150)			5.426 (7,148)		

* *p* < 0.05, **p < 0.01, ***p < 0.001.

*Note:* Model 1 includes FGF19 as the independent variable and BDI scores as the dependent variable. Model 2 includes smoking status as the independent variable and BDI scores as the dependent variable. Model 3 includes FGF19, smoking, and their interaction term (FGF19 × smoking) as independent variables. All models were adjusted for age, BMI, marital status, and living status. All analyses were conducted as moderation analyses.

**Abbreviations:** FGF19, fibroblast growth factor 19; BMI, body mass index; BDI, Beck’s Depression Inventory-II.

**Figure 2.**
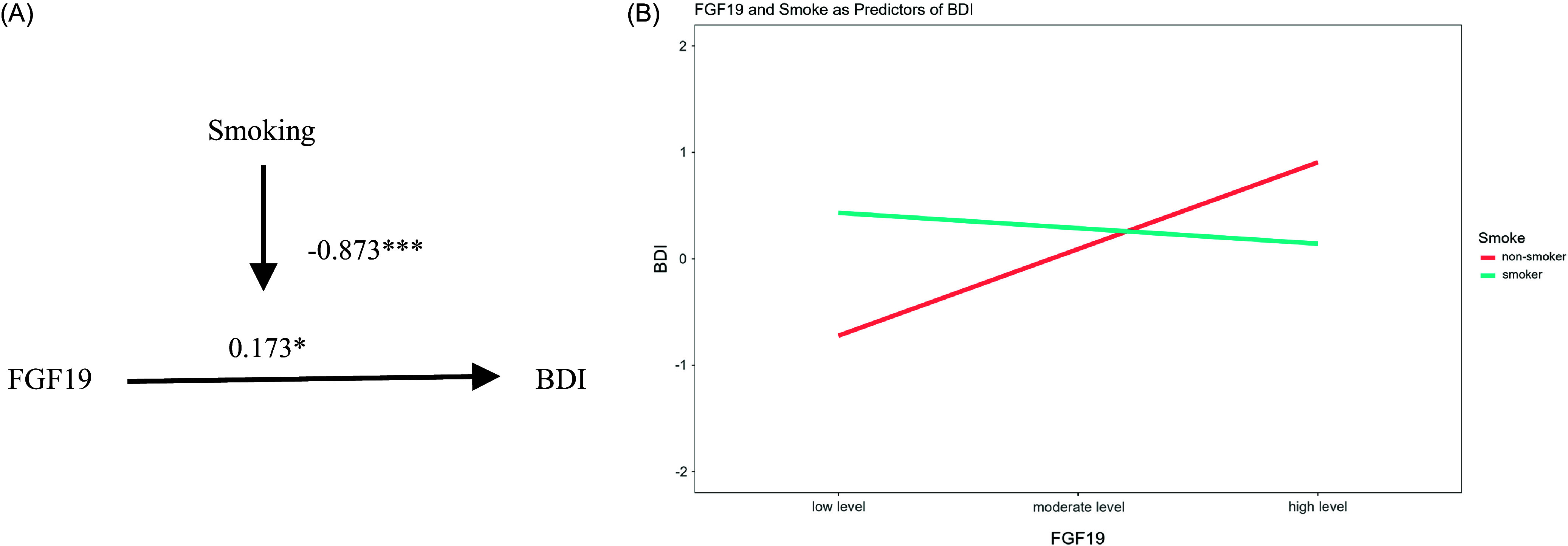
Moderation effect of smoking on FGF19 and BDI scores. *Note*: The two regression lines represent the association between FGF19 and BDI scores in non-smokers and smokers.**Abbreviations:** FGF19, fibroblast growth factor 19; BDI, Beck’s Depression Inventory-II.

Multiple linear regression analyses indicated that both FGF19 and smoking were independently associated with BDI scores. Specifically, FGF19 was positively associated with BDI scores (*β* = 0.173, 95% CI: 0.015–0.331, *t* = 2.161, *p* < 0.05, adj. R^2^ = 0.033), and smoking also showed a significant positive association (*β* = 0.444, 95% CI: 0.120–0.769, *t* = 2.708, *p* < 0.01, adj. R^2^ = 0.049) in Model 1 and Model 2, respectively (Table 2). In Model 3, we included the interaction term between FGF19 and smoking. Results showed a significant interaction effect (*β* = −0.873, 95% CI: −1.244 to −0.501, *t* = −4.644, *p* < 0.001, adj. R^2^ = 0.167), indicating that smoking moderated the relationship between FGF19 and BDI scores (Table 2; Figure 2A).

The moderating effect of smoking was further supported by an increase in the F-value from 2.062 in Model 1 to 5.426 in Model 3 (ΔF = 3.364, *p* < 0.001). To clarify this moderation effect, we performed a simple slopes analysis to explore the association between FGF19 and BDI scores within non-smokers and smokers (Table 3; Figure 2B). Among non-smokers, FGF19 showed a significant positive association with BDI scores (*β* = 0.741, 95% CI: 0.424–1.058, *t* = 4.618, *p* < 0.001), whereas in smokers, this association was not statistically significant (*β* = −0.132, 95% CI: −0.322–0.058, *t* = −1.372, *p* = 0.172).

**Table 3.** Simple slopes analysis

Smoke	*β*	*t*	*p*	95%CI
Non-smoker	0.741	4.618	< 0.001	[0.424, 1.058]
Smoker	−0.132	−1.372	0.172	[−0.322, 0.058]

## Discussion

Our study indicates that cigarette smoking is positively associated with CSF FGF19 levels and depressive symptoms as assessed by BDI scores (see Table 1). Moreover, we found a positive association between CSF FGF19 levels and BDI scores in non-smokers, while this effect was absent in smokers, consistent with our previous research (Liu *et al*., 2017). Further analysis using moderation models indicated that smoking inhibits the association between FGF19 and depressive symptoms, exerting a negative moderation effect.

We found that CSF FGF19 levels increased in individuals with higher BDI scores, suggesting that FGF19 may play a significant role in depressive symptoms. FGF19, a member of the fibroblast growth factor (FGF) family, is known to influence brain development (Nishimura *et al*., 1999; Somm & Jornayvaz, 2018). For instance, the mouse ortholog FGF15 has been shown to exert neuroprotective effects against oxidative stress (Zhang *et al*., 2019). FGF15 has also been implicated in depression via the Farnesoid X Receptor (FXR) signalling axis (Cai *et al*., 2023; Wang *et al*., 2025). Consistent with our findings, Tang and colleagues (2023) reported a positive association between plasma FGF19 levels and BDI in patients with MDD and proposed that fluctuations in FGF19 might contribute to the metabolic and cognitive disturbances observed in MDD patients (Tang *et al*., 2023). FGF19 is known to regulate bile acid metabolism, and BAs have neuroprotective effects, enhancing BDNF release and stimulating the BDNF–TrkB pathway (Li *et al*., 2020; Zhai *et al*., 2023). Numerous studies have indicated that BDNF has potential antidepressant effects (Zhang *et al*., 2016; Phillips, 2017; Zhang & Liao, 2020). A clinical study demonstrated that individuals with the BDNF val66met genotype exhibited reduced BDNF secretion, deficits in situational memory function, and an increased risk of anxiety and depression (Egan *et al*., 2003; Hariri *et al*., 2003). Intracerebroventricular injection of FGF19 in mice has been shown to suppress HPA axis activity (Perry *et al*., 2015), suggesting that reduced FGF19 may impair stress regulation and contribute to depressive symptoms.

Smoking is known to impair glucose metabolism and insulin sensitivity (Chiolero *et al*., 2008), which may in turn, influence FGF19 signalling. FGF19 plays a critical role in bile acid synthesis and glucose homeostasis (Potthoff *et al*., 2011, Degirolamo *et al*., 2016). Disruption of the FXR–FGF19 axis has been implicated in metabolic disorders such as inflammatory bowel disease (IBD), obesity, and type 2 diabetes (Gadaleta *et al*., 2020; Bag Soytas *et al*., 2021; Lyutakov *et al*., 2021). Reduced circulating FGF19 levels in patients with active IBD and obesity further support its involvement in maintaining metabolic balance (Schreuder *et al*., 2010; Mráz *et al*., 2011). Thus, smoking-induced metabolic dysregulation may impair FGF19 function and thereby modulate its association with depressive symptoms (Wang *et al*., 2024).

One possible explanation is that chronic exposure to inflammation, as seen in smokers, may create an immunologically ‘tolerant’ state (Esquivel *et al*., 2014). Smoking is a known pro-inflammatory factor that elevates cytokines such as TNF-α and IL-6 (Sanada *et al*., 2018; Womack & Justice, 2020), and inflammation has been shown to downregulate FGF15 expression in animal models (Gadaleta *et al*., 2020). However, the specific mechanisms through which nicotine influences central nervous system metabolism and inflammation to impact depression remain unclear and warrant further investigation. Our findings contribute to this area by suggesting that smoking may interfere with the regulatory role of CSF FGF19 in depressive symptomatology.

Based on our findings, we hypothesise that elevated BDI scores may be linked to increased FGF19 levels, as a compensatory mechanism to counteract depressive symptoms, potentially via modulation of the BDNF–TrkB axis or HPA axis. However, this relationship was not observed in smokers, suggesting that smoking status could modulate the interaction between FGF19 and depressive symptoms. Further research is needed to elucidate the precise role of smoking in this context. This finding is consistent with our previous research, which also found that CSF FGF19 was positively correlated with BDI scores (Liu *et al*., 2017). Smoking is widely recognised as a detrimental lifestyle habit (Tsai *et al*., 2019; Wiegman *et al*., 2020; Ma *et al*., 2021). Therefore, we hypothesise that this may be one reason for the negative impact of smoking on the association between FGF19 and BDI scores.

To our knowledge, this is the first study to assess the role of smoking on FGF19-induced depressive symptoms (expressed as BDI) in Chinese men. Our findings indicate that the positive effects of CSF FGF19 on BDI are negatively impacted by smoking.

However, this study has several limitations. First, causal inferences cannot be drawn from a case-control design, and the small sample size may limit the statistical power to examine associations and moderation. Therefore, evidence from prospective studies with larger sample sizes is warranted. Second, retrospective recall bias may occur with the use of subjective depression measures and smoking assessments. Additionally, other potential confounders may affect our understanding of the relationship between smoking and depressive disorders. Finally, the lower prevalence of smoking in women and the recruitment of only men result in limited applicability and generalizability.

## Conclusion

These findings support a potential role of FGF19 in depression and highlight the importance of considering smoking status when evaluating this association. The findings of this study have important clinical implications. First, they emphasise the need to consider smoking when assessing the relationship between FGF19 and depressive symptoms. Clinicians may need to integrate smoking cessation strategies into treatment plans for patients with depressive symptoms who also use tobacco. Future research should explore potential mechanisms and develop effective interventions. Overall, these results highlight the potential role of FGF19 in individuals at risk for presence of depressive symptoms and underscore the importance of considering smoking status when examining this association.

## Supporting information

Li et al. supplementary material 1 Li et al. supplementary material

Li et al. supplementary material 2 Li et al. supplementary material

## Data Availability

The raw data supporting the conclusions of this article will be made available by the authors on request.

## Supplementary material

The supplementary material for this article can be found at https://doi.org/10.1017/neu.2025.10028.

## Inclusion of identifiable human data

No potentially identifiable human images or data is presented in this study.

## References

[ref1] Bag Soytas R , Suzan V , Arman P , Emiroglu Gedik T , Unal D , Cengiz M , Bolayirli IM , Suna Erdincler D , Doventas A and Yavuzer H (2021) Association of FGF-19 and FGF-21 levels with primary sarcopenia. Geriatrics & Gerontology International 21(10), 959–962. 34405516 10.1111/ggi.14263

[ref2] Beenken A and Mohammadi M (2009) The FGF family: biology, pathophysiology and therapy. Nature Reviews Drug Discovery 8(3), 235–253. 19247306 10.1038/nrd2792 PMC3684054

[ref3] Cai W , Li C , Su Z , Cao J , Chen Z , Chen Y , Guo Z , Cai J and Xu F (2023) Profile of the bile acid FXR-FGF15 pathway in the glucolipid metabolism disorder of diabetic mice suffering from chronic stress. PeerJ 11, e16407. 38025699 10.7717/peerj.16407 PMC10656902

[ref4] Castrén E and Monteggia LM (2021) Brain-derived neurotrophic factor signaling in depression and antidepressant action. Biological Psychiatry 90(2), 128–136. 34053675 10.1016/j.biopsych.2021.05.008

[ref5] Chiolero A , Faeh D , Paccaud F and Cornuz J (2008) Consequences of smoking for body weight, body fat distribution, and insulin resistance. The American Journal of Clinical Nutrition 87(4), 801–809. 18400700 10.1093/ajcn/87.4.801

[ref6] Coultas DB , Edwards DW , Barnett B and Wludyka P (2007) Predictors of depressive symptoms in patients with COPD and health impact. COPD: Journal of Chronic Obstructive Pulmonary Disease 4(1), 23–28. 17364674 10.1080/15412550601169190

[ref7] Degirolamo C , Sabbà C and Moschetta A (2016) Therapeutic potential of the endocrine fibroblast growth factors FGF19, FGF21 and FGF23. Nature Reviews Drug Discovery 15(1), 51–69. 26567701 10.1038/nrd.2015.9

[ref8] Deng Z , Deng S , Zhang MR and Tang MM (2019) Fibroblast growth factors in depression. Frontiers in Pharmacology 10, 60. 30804785 10.3389/fphar.2019.00060 PMC6370647

[ref9] Dierker L and Donny E (2008) The role of psychiatric disorders in the relationship between cigarette smoking and DSM-IV nicotine dependence among young adults. Nicotine & Tobacco Research 10(3), 439–446. 18324562 10.1080/14622200801901898 PMC2679531

[ref10] Egan MF , Kojima M , Callicott JH , Goldberg TE , Kolachana BS , Bertolino A , Zaitsev E , Gold B , Goldman D , Dean M , Lu B and Weinberger DR (2003) The BDNF val66met polymorphism affects activity-dependent secretion of BDNF and human memory and hippocampal function. Cell 112(2), 257–269. 12553913 10.1016/s0092-8674(03)00035-7

[ref11] Esquivel AL , Pérez-Ramos J , Cisneros J , Herrera I , Rivera-Rosales R , Montaño M and Ramos C (2014) The effect of obesity and tobacco smoke exposure on inflammatory mediators and matrix metalloproteinases in rat model. Toxicology Mechanisms and Methods 24(9), 633–643. 25141943 10.3109/15376516.2014.956911

[ref12] Fluharty M , Taylor AE , Grabski M and Munafò MR (2017) The association of cigarette smoking with depression and anxiety: a systematic review. Nicotine & Tobacco Research 19(1), 3–13. 27199385 10.1093/ntr/ntw140 PMC5157710

[ref13] Gadaleta RM , Garcia-Irigoyen O , Cariello M , Scialpi N , Peres C , Vetrano S , Fiorino G , Danese S , Ko B , Luo J , Porru E , Roda A , Sabbà C and Moschetta A (2020) Fibroblast growth factor 19 modulates intestinal microbiota and inflammation in presence of Farnesoid X Receptor. EBioMedicine 54, 102719. 32259714 10.1016/j.ebiom.2020.102719 PMC7136604

[ref14] Gadaleta RM and Moschetta A (2019) Metabolic messengers: fibroblast growth factor 15/19. Nature Metabolism 1(6), 588–594. 10.1038/s42255-019-0074-3 32694803

[ref15] Gravely-Witte S , Stewart DE , Suskin N and Grace SL (2009) The association among depressive symptoms, smoking status and antidepressant use in cardiac outpatients. Journal of Behavioral Medicine 32(5), 478–490. 19504177 10.1007/s10865-009-9218-3 PMC2927523

[ref16] Hall SM , Muñoz RF , Reus VI and Sees KL (1993) Nicotine, negative affect, and depression. Journal of Consulting and Clinical Psychology 61(5), 761–767. 7902368 10.1037//0022-006x.61.5.761

[ref17] Hariri AR , Goldberg TE , Mattay VS , Kolachana BS , Callicott JH , Egan MF and Weinberger DR (2003) Brain-derived neurotrophic factor val66met polymorphism affects human memory-related hippocampal activity and predicts memory performance. The Journal of Neuroscience 23(17), 6690–6694. 12890761 10.1523/JNEUROSCI.23-17-06690.2003 PMC6740735

[ref18] Haustein KO , Haffner S and Woodcock BG (2002) A review of the pharmacological and psychopharmacological aspects of smoking and smoking cessation in psychiatric patients. International Journal of Clinical Pharmacology and Therapeutics 40(09), 404–418. 12358157 10.5414/cpp40404

[ref19] Hooshmand S , Willoughby T and Good M (2012) Does the direction of effects in the association between depressive symptoms and health-risk behaviors differ by behavior? A longitudinal study across the high school years. Journal of Adolescent Health 50(2), 140–147. 10.1016/j.jadohealth.2011.05.016 22265109

[ref20] Laarne PH , Tenhunen-Eskelinen ML , Hyttinen JK and Eskola HJ (2000) Effect of EEG electrode density on dipole localization accuracy using two realistically shaped skull resistivity models. Brain Topography 12(4), 249–254. 10912732 10.1023/a:1023422504025

[ref21] Lang UE and Borgwardt S (2013) Molecular mechanisms of depression: perspectives on new treatment strategies. Cellular Physiology and Biochemistry 31(6), 761–777. 23735822 10.1159/000350094

[ref22] Lawrence D , Mitrou F and Zubrick SR (2009) Smoking and mental illness: results from population surveys in Australia and the United States. BMC Public Health 9(1), 285. 19664203 10.1186/1471-2458-9-285 PMC2734850

[ref23] Li C , Wang X , Yan J , Cheng F , Ma X , Chen C , Wang W and Wang Q (2020) Cholic acid protects in vitro neurovascular units against oxygen and glucose deprivation-induced injury through the BDNF-TrkB signaling pathway. Oxidative Medicine and Cellular Longevity 2020, 1201624–14. 33101581 10.1155/2020/1201624 PMC7576336

[ref24] Li H , Chen J , Chen C , Xu Z , Xu J , Lin W , Wu J , Li G , Xu H , Kang Y , Wang F and Liu Y (2018) CSF glutamate level decreases in heavy smokers and negatively correlates with BDI scores. Psychiatry Research 270, 627–630. 30384282 10.1016/j.psychres.2018.10.053

[ref25] Li Y , Wu F , Mu Q , Xu K , Yang S , Wang P , Wu Y , Wu J , Wang W , Li H , Chen L , Wang F and Liu Y (2022) Metal ions in cerebrospinal fluid: associations with anxiety, depression, and insomnia among cigarette smokers. CNS Neuroscience & Therapeutics 28(12), 2141–2147. 36168907 10.1111/cns.13955 PMC9627395

[ref26] Liu Y , Yu D , Wang X , Tan X and Wang F (2017) Is cerebrospinal fluid fibroblast growth factor 19 (FGF19) a mood regulator? Neuropsychiatry 07(02), 126–130.

[ref27] Lyutakov I , Nakov R , Valkov H , Vatcheva-Dobrevska R , Vladimirov B and Penchev P (2021) Serum levels of fibroblast growth factor 19 Correlate with the severity of diarrhea and independently from intestinal inflammation in patients with inflammatory bowel disease or microscopic colitis. The Turkish Journal of Gastroenterology 32(4), 374–381. 34231484 10.5152/tjg.2021.20247 PMC8975464

[ref28] Ma Y , Long Y and Chen Y (2021) Roles of inflammasome in cigarette smoke-related diseases and physiopathological disorders: mechanisms and therapeutic opportunities. Frontiers in Immunology 12, 720049. 34367189 10.3389/fimmu.2021.720049 PMC8334727

[ref29] Makarov PO (1973) Effect of ultrasound on time characteristics of human skin reception. Fiziologicheskii zhurnal SSSR imeni I. M. Sechenova 59(1), 39–43. 4684675

[ref30] Mcclave AK , Mcknight-Eily LR , Davis SP and Dube SR (2010) Smoking characteristics of adults with selected lifetime mental illnesses: results from the 2007 National health interview survey. American Journal of Public Health 100(12), 2464–2472. 20966369 10.2105/AJPH.2009.188136 PMC2978196

[ref31] Monroe SM and Harkness KL (2022) Major depression and its recurrences: life course matters. Annual Review of Clinical Psychology 18(1), 329–357. 10.1146/annurev-clinpsy-072220-021440 35216520

[ref32] Mráz M , Lacinová Z , Kaválková P , Haluzíková D , Trachta P , Drápalová J , Hanušová V and Haluzík M (2011) Serum concentrations of fibroblast growth factor 19 in patients with obesity and type 2 diabetes mellitus: the influence of acute hyperinsulinemia, very-low calorie diet and PPAR-α agonist treatment. Physiological Research 60, 627–636. 21574752 10.33549/physiolres.932099

[ref33] Nishimura T , Utsunomiya Y , Hoshikawa M , Ohuchi H and Itoh N (1999) Structure and expression of a novel human FGF, FGF-19, expressed in the fetal brain. Biochimica et Biophysica Acta (BBA) - Gene Structure and Expression 1444(1), 148–151. 9931477 10.1016/s0167-4781(98)00255-3

[ref34] Orth SR (2002) Cigarette smoking: an important renal risk factor - far beyond carcinogenesis. Tobacco Induced Diseases 1(2), 137–155. 19570254 10.1186/1617-9625-1-2-137 PMC2671650

[ref35] Perry RJ , Lee S , Ma L , Zhang D , Schlessinger J and Shulman GI (2015) FGF1 and FGF19 reverse diabetes by suppression of the hypothalamic-pituitary-adrenal axis. Nature Communications 6(1), 6980. 10.1038/ncomms7980 PMC4413509 25916467

[ref36] Phillips C (2017) Brain-derived neurotrophic factor, depression, and physical activity: making the neuroplastic connection. Neural Plasticity 2017, 7260130. 28928987 10.1155/2017/7260130 PMC5591905

[ref37] Picciotto MR , Brunzell DH and Caldarone BJ (2002) Effect of nicotine and nicotinic receptors on anxiety and depression. Neuroreport 13(9), 1097–1106. 12151749 10.1097/00001756-200207020-00006

[ref38] Potthoff MJ , Boney-Montoya J , Choi M , He T , Sunny NE , Satapati S , Suino-Powell K , Xu HE , Gerard RD , Finck BN , Burgess SC , Mangelsdorf DJ and Kliewer SA (2011) FGF15/19 regulates hepatic glucose metabolism by inhibiting the CREB-PGC-1α pathway. Cell Metabolism 13(6), 729–738. 21641554 10.1016/j.cmet.2011.03.019 PMC3131185

[ref39] Richards CS , Cohen LM , Morrell HE , Watson NL and Low BE (2013) Treating depressed and anxious smokers in smoking cessation programs. Journal of Consulting and Clinical Psychology 81(2), 263–273. 22428940 10.1037/a0027793

[ref40] Ríos-Bedoya CF , Snedecor SM , Pomerleau CS and Pomerleau OF (2008) Association of withdrawal features with nicotine dependence as measured by the Fagerström test for nicotine dependence (FTND). Addictive Behaviors 33(8), 1086–1089. 18502052 10.1016/j.addbeh.2008.04.005 PMC2553560

[ref41] Sanada F , Taniyama Y , Muratsu J , Otsu R , Shimizu H , Rakugi H and Morishita R (2018) Source of chronic inflammation in aging. Frontiers in Cardiovascular Medicine 5, 12. 29564335 10.3389/fcvm.2018.00012 PMC5850851

[ref42] Schreuder TC , Marsman HA , Lenicek M , Van Werven JR , Nederveen AJ , Jansen PL and Schaap FG (2010) The hepatic response to FGF19 is impaired in patients with nonalcoholic fatty liver disease and insulin resistance. American Journal of Physiology-Gastrointestinal and Liver Physiology 298(3), G440–5. 20093562 10.1152/ajpgi.00322.2009

[ref43] Somm E and Jornayvaz FR (2018) Fibroblast growth factor 15/19:From basic functions to therapeutic perspectives. Endocrine Reviews 39(6), 960–989. 30124818 10.1210/er.2018-00134

[ref44] Sweitzer MM , Donny EC , Dierker LC , Flory JD and Manuck SB (2008) Delay discounting and smoking: association with the Fagerström test for nicotine dependence but not cigarettes smoked per day. Nicotine & Tobacco Research 10(10), 1571–1575. 18946776 10.1080/14622200802323274

[ref45] Tan Q , Li F , Wang G , Xia W , Li Z , Niu X , Ji W , Yuan H , Xu Q , Luo Q , Zhang J and Lu S (2016) Identification of FGF19 as a prognostic marker and potential driver gene of lung squamous cell carcinomas in Chinese smoking patients. Oncotarget 7(14), 18394–18402. 26943773 10.18632/oncotarget.7817 PMC4951296

[ref46] Tang M , Cheng S , Wang L , Tang H , Liu T , Zhao T and Dang R (2023) Decreased FGF19 and FGF21: possible underlying common pathogenic mechanism of metabolic and cognitive dysregulation in depression. Frontiers in Neuroscience 17, 1165443. 37266540 10.3389/fnins.2023.1165443 PMC10229787

[ref47] Tran BX , Ha GH , Nguyen DN , Nguyen TP , Do HT , Latkin CA , Ho CSH and Ho RCM (2020) Global mapping of interventions to improve quality of life of patients with depression during 1990–2018. Quality of Life Research 29(9), 2333–2343. 32347440 10.1007/s11136-020-02512-7

[ref48] Tsai M , Song MA , Mcandrew C , Brasky TM , Freudenheim JL , Mathé E , Mcelroy J , Reisinger SA , Shields PG and Wewers MD (2019) Electronic versus combustible cigarette effects on inflammasome component release into human lung. American Journal of Respiratory and Critical Care Medicine 199(7), 922–925. 30608866 10.1164/rccm.201808-1467LE PMC6444658

[ref49] Turner CA , Akil H , Watson SJ and Evans SJ (2006) The fibroblast growth factor system and mood disorders. Biological Psychiatry 59(12), 1128–1135. 16631131 10.1016/j.biopsych.2006.02.026

[ref50] Wang CS , Kavalali ET and Monteggia LM (2022) BDNF signaling in context: from synaptic regulation to psychiatric disorders. Cell 185(1), 62–76. 34963057 10.1016/j.cell.2021.12.003 PMC8741740

[ref51] Wang G , Hallberg J , Hernandez-Pacheco N , Ekström S , Vercalsteren E , Brew BK , Almqvist C , Janson C , Kull I , Bergström A , Melén E and Lu D (2024) Depression in childhood to early adulthood and respiratory health in early adulthood. BJPsych Open 10(6), e202. 39523673 10.1192/bjo.2024.794 PMC11698180

[ref52] Wang W , Bai X , Li J , Wang S , Zhao F , Qin X and Gao X (2025) Low polarity fraction of Radix Bupleuri alleviates chronic unpredictable mild stress-induced depression in rats through FXR modulating bile acid homeostasis in liver, gut, and brain. Journal of Pharmaceutical and Biomedical Analysis 253, 116523. 39489929 10.1016/j.jpba.2024.116523

[ref53] Wiegman CH , Li F , Ryffel B , Togbe D and Chung KF (2020) Oxidative stress in Ozone-induced chronic lung inflammation and emphysema: a facet of chronic obstructive pulmonary disease. Frontiers in Immunology 11, 1957. 32983127 10.3389/fimmu.2020.01957 PMC7492639

[ref54] Wojnowski L and Zimmer A (1997) Use of transgenic mice to study activation of retinoic acid-responsive promoters. Methods in Enzymology 282, 77–85. 9330278 10.1016/s0076-6879(97)82097-6

[ref55] Womack JA and Justice AC (2020) The OATH Syndemic: opioids and other substances, aging, alcohol, tobacco, and HIV. Current Opinion in HIV and AIDS 15(4), 218–225. 32487817 10.1097/COH.0000000000000635 PMC7422477

[ref56] Wootton RE , Richmond RC , Stuijfzand BG , Lawn RB , Sallis HM , Taylor GMJ , Hemani G , Jones HJ , Zammit S , Davey Smith G and Munafò MR (2020) Evidence for causal effects of lifetime smoking on risk for depression and schizophrenia: a Mendelian randomisation study. Psychological Medicine 50(14), 2435–2443. 31689377 10.1017/S0033291719002678 PMC7610182

[ref57] Xu J , Li H , Wang F , Xu Z , Li G , Ding C , Wu J , Kang Y , Li H , Xu H and Liu Y (2019) Lack of correlation between CSF glutamate levels and PSQI scores in heavy smokers. Sleep and Breathing 23(1), 297–302. 30088240 10.1007/s11325-018-1705-8

[ref58] Xu YH , Zhu Y , Zhu YY , Wei H , Zhang NN , Qin JS , Zhu XL , Yu M and Li YF (2021) Abnormalities in FGF family members and their roles in modulating depression‐related molecules. European Journal of Neuroscience 53(1), 140–150. 31491043 10.1111/ejn.14570

[ref59] Zhai W , Zhang T , Jin Y , Huang S , Xu M and Pan J (2023) The fibroblast growth factor system in cognitive disorders and dementia. Frontiers in Neuroscience 17, 1136266. 37214403 10.3389/fnins.2023.1136266 PMC10196031

[ref60] Zhang E and Liao P (2020) Brain-derived neurotrophic factor and post-stroke depression. Journal of Neuroscience Research 98(3), 537–548. 31385340 10.1002/jnr.24510

[ref61] Zhang JC , Yao W and Hashimoto K (2016) Brain-derived neurotrophic factor (BDNF)-TrkB signaling in inflammation-related depression and potential therapeutic targets. Current Neuropharmacology 14(7), 721–731. 26786147 10.2174/1570159X14666160119094646 PMC5050398

[ref62] Zhang Z , Wang N , Zhang Y , Zhao J and Lv J (2019) Downregulation of microRNA-302b-3p relieves oxygen-glucose deprivation/re-oxygenation induced injury in murine hippocampal neurons through up-regulating Nrf2 signaling by targeting fibroblast growth factor 15/19. Chemico-Biological Interactions 309, 108705. 31199929 10.1016/j.cbi.2019.06.018

