# Supplementary material for: Association between fibroblast growth factor 19 and depressive symptoms: the moderating role of smoking
Source: Acta Neuropsychiatr. 2025 Jul 14;37:e74. doi: 10.1017/neu.2025.10028 (PMC13130274; doi:10.1017/neu.2025.10028)
Supplement: Li et al. supplementary material 1 — Li et al. supplementary material [file S0924270825100288sup001.docx]

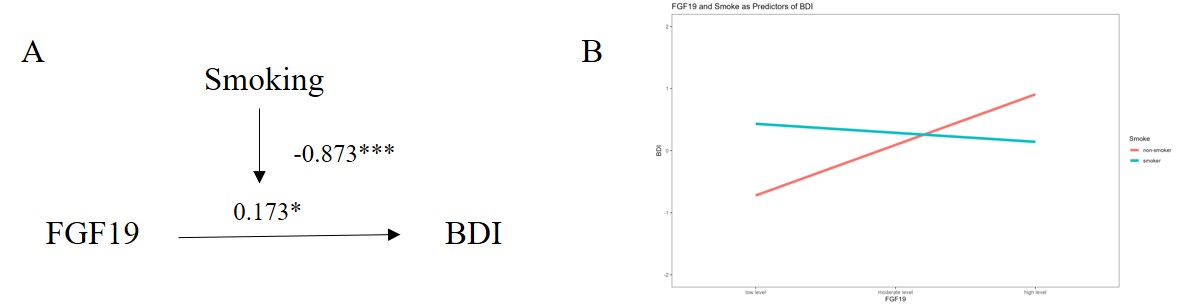


Methods

Conclusions

Results


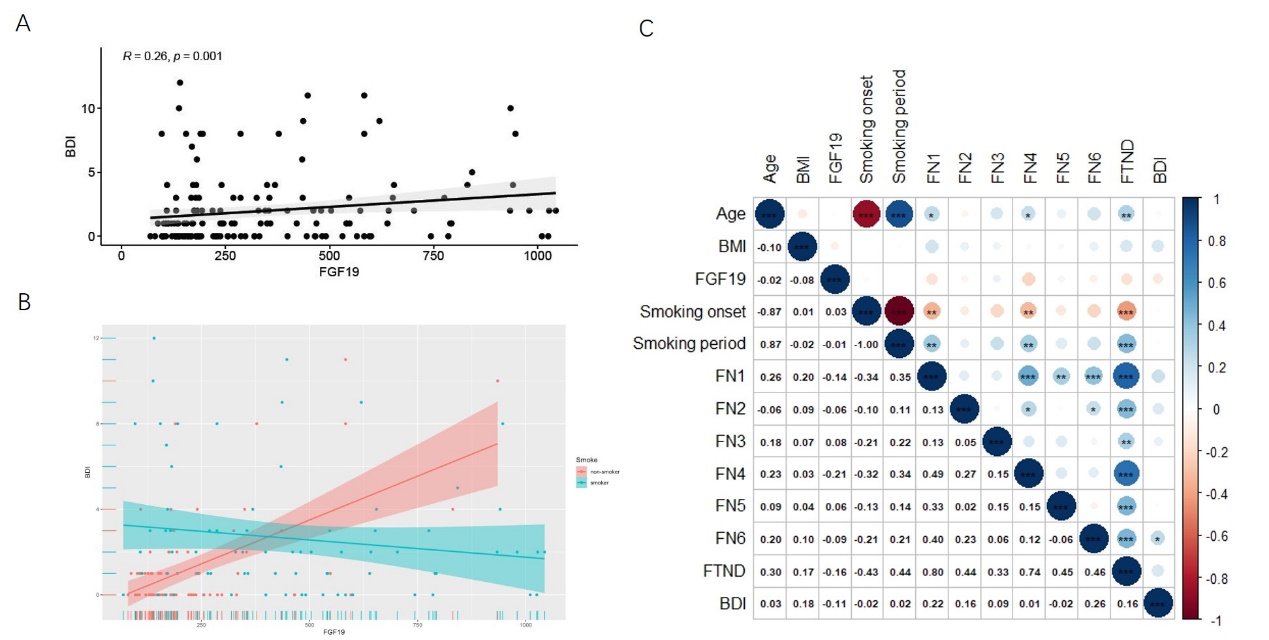

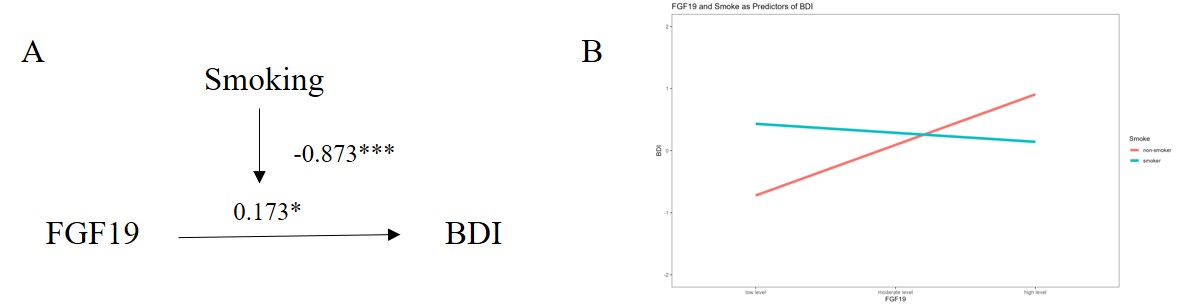


- 156 Chinese adult males

(78 smokers and 78 non-smokers) and severity of depressive symptoms was evaluated using the BDI scores.

- Spearman rank correlation

analyses were used to

investigate the relationship between cerebrospinal fluid (CSF) FGF19 levels and BDI scores.

- Additionally,moderation

and simple slope analyses were applied to assess the

moderating effect of smoking on the relationship between the two.

- Figure 1. Correlation analysis between FGF19 and BDI scores.
- Figure 2. Moderation effect of smoking on FGF19 and BDI scores.

These results highlight the potential role of FGF19 in individuals at risk for presence of or further development of depressive symptoms and underscore the importance of considering smoking status when examining this association.
