# Supplementary material for: Association between fibroblast growth factor 19 and depressive symptoms: the moderating role of smoking
Source: Acta Neuropsychiatr. 2025 Jul 14;37:e74. doi: 10.1017/neu.2025.10028 (PMC13130274; doi:10.1017/neu.2025.10028)
Supplement: Li et al. supplementary material 2 — Li et al. supplementary material [file S0924270825100288sup002.docx]

**Supplementary Table 1 Basic information between the groups.**

|  | **Little to no risk for presence of depressive symptoms** | **At risk for presence of depressive symptoms** | **Mild depressive symptoms** | **Moderate and severe depressive symptoms** | ***p*-value** |
| --- | --- | --- | --- | --- | --- |
|  | **（BDI scores < 1 (n=63)）** | **(1 ≤ BDI scores < 5 (n=74))** | **(5 ≤ BDI scores < 8 (n=5))** | **(BDI scores > 8 (n=14))** |  |
| Age | 28.7 (8.6) | 33.8 (10.5) | 39.8 (14.8) | 32.3 (9.5) | 0.016 |
| BMI | 25.1 (3.8) | 25.4 (3.9) | 29.5 (5.8) | 26.0 (4.1) | 0.350 |
| FGF19 | 278.4 (218.0) | 370.1 (258.1) | 406.8 (315.1) | 408.8 (279.1) | 0.051 |
| BDI scores | 0 (0) | 2.0 (1.0) | 6.0 (0.8) | 9.1 (1.4) | < 0.001 |

**Supplementary Table 2 Comparisons between non-smokers and smokers (presentation of the median and quartile of the non-normal distribution).**

| **Characteristic** | **Non-smokers,** | **Smokers,** | ***p*-value**^2^ |
| --- | --- | --- | --- |
|  | **N = 78^1^** | **N = 78^1^** |  |
| Age | 27.0 [21.0;37.0] | 32.0 [27.0;37.0] | 0.014 |
| BMI | 24.2 [22.3;26.7] | 25.6 [23.4;28.3] | 0.036 |
| FGF19 (pg/ml) | 177.0 [130.7;252.6] | 410.0 [188.5;614.8] | <0.001 |
| Smoking onset |  | 20.0 [18.0;22.0] |  |
| Smoking period |  | 10.5 [7.0;17.5] |  |
| FTND |  | 4.0 [1.8;5.0] |  |
| BDI | 0 [0;1.0] | 2.0 [1.0;3.0] | <0.001 |
